# Impact of foreign players’ participation in the Palestinian basketball league on team cohesion from the perspective of local players

**DOI:** 10.3389/fpsyg.2025.1545018

**Published:** 2025-02-19

**Authors:** Iyad A. Yousef, Mahmoud W. Kayed, Sana M. Liftawi

**Affiliations:** Department of Physical Education, College of Education, Birzeit University, Ramallah, Palestine

**Keywords:** foreign players, Palestine basketball league, team cohesion, local players, team performance

## Abstract

**Objective:**

The purpose of this paper is to assess the impact of foreign players’ participation in the Palestinian Basketball League on team cohesion from the perspective of local players.

**Methods:**

Using a descriptive-analytical method, the researchers developed a cohesion measurement tool with three dimensions: psychological, social, and team belonging. The tool comprised 30 items, equally distributed across the dimensions, based on player status, experience, and position. The study surveyed 84 players aged between 18 and 38 in the Palestinian Basketball League.

**Results:**

The findings revealed that cohesion was rated as moderate (mean = 3.32), with higher scores in team affiliation (3.50) compared to psychological cohesion (3.21). Experience-based differences showed that players with less than 5 years and more than 10 years of experience exhibited higher cohesion levels compared to those with 6–10 years. Positional differences favored forwards and guards. Regarding player status, reserve players demonstrated higher cohesion in the psychological and team belonging dimensions, although no significant differences were found in the social dimension.

**Conclusion:**

The study concludes that foreign players have a positive impact on team cohesion, and it was rated as moderate with higher scores in team affiliation compared to psychological cohesion. The researchers recommend careful selection of foreign players to enhance team cohesion and stability. This study contributes to the understanding of foreign players’ role in fostering team dynamics and performance in the Palestinian Basketball League, expanding the literature on the topic. This study is original and valuable, as there has been almost no previous research that has measured and evaluated the impact of foreign players’ participation in the Palestinian basketball league on team cohesion from the perspective of local players.

## Introduction

Sports are an economic and social phenomenon. It takes a multi-scientific approach originating from several research domains to fully understand. Fans become interested in sports when they see something thrilling even if they do not know how it will end. This is only true if there is an unexpected result and both teams are of roughly similar strength. Basketball in Palestine is known as the sport that has gained widespread acceptance and interest from fans. It is organized in recent tournaments and national and international leagues. The presence of foreign players in national Arab basketball leagues and tournaments is a fundamental pillar on which teams rely to develop and elevate the level of Arab basketball. These players possess different technical attributes and qualities compared to local players. The goal of recruiting them in most Arab countries that prioritize basketball, such as Lebanon, Jordan, Iraq, the UAE, and Saudi Arabia, is to enhance the level of local players, motivate them, and facilitate the exchange of experiences. Additionally, the presence of foreign players increases spectator turnout and enthusiasm among fans. Their presence also helps develop interactions among players, thereby contributing to the improvement of national teams and providing valuable experience, especially for young and emerging players. Foreign players serve as role models and motivators, particularly for younger players.

Team cohesion is a fundamental concept in sports psychology, defined as the dynamic process that binds individuals in a team and motivates them to work together toward shared goals. According to classical literature, such as Carron et al. (1985), cohesion consists of two main dimensions: social cohesion and task cohesion. These dimensions highlight the relationships among team members and their ability to collaborate effectively to achieve performance objectives.

Maguire and Poulton (1999) examine how national identity, sport, and the media interact in the English press during EURO 96, the European Football Championship. In light of current European politics, the study aims to draw attention to certain media portrayals that surround and support sport in general and (association) football in particular. A qualitative discourse analysis of some of the English newspaper coverage of EURO 96 is the main method used to accomplish this. The English position in relation to their/its continental European neighbors will be taken into consideration as they analyze the identity politics that were on display during the championships. More generally, they aim to argue that Elias’s analysis of the socio-genesis of more deeply ingrained national character and habitus codes also provides significant insight into contemporary European identity politics, even though the ideas of imagined communities and “invented traditions” are helpful in understanding identity politics. The article’s foundation is thus a particular method of examining sport and national identity that comes from a process-sociological viewpoint.

Sports labor migration around the world is discussed by Maquire and Falcous (2005). First reported by Maguire (1988), the contentious presence of North American basketball players in English is examined in relation to concerns about how local cultures view immigrants. Fans’ consumption of local basketball was examined in a two-year ethnographic project that included focus groups, interviews, and participant observations. Interpretations of migrant players were varied and complex. These were influenced by local identities and civic pride, as well as by local spectator experiences and cultural stereotypes. As a result, the local perspective is used to specifically analyze the presence of migrant athletes. The diverse functions and meanings of basketball consumption in the lives of the local population influenced the responses. These findings support the need for case studies with an empirical foundation to examine local consumption in the context of the larger political-economic trends in international sport.

Poulton (2004) investigates the connection between media sport and national identity, paying particular attention to how national identities were constructed and portrayed during the 1996 European Football Championships (Euro ‘96) broadcast. Although Hobsbawm’s and Anderson’s ideas of “imagined communities” and “invented traditions,” respectively, are helpful in this context, the process-sociological approach is also very beneficial. It has been demonstrated that the basis of cultural relations, identity politics, and the creation and portrayal of national identities are significantly influenced by national habitus codes, embodied emotions, and the practical consciousness of the people who make up a nation. According to the findings of this textual analysis of Euro ‘96 television coverage, which are backed up by knowledge of the production codes that were used to create the texts, the English were more emotionally identified with their own country than with the we-image of being also Europeans. The results imply that international sporting events such as Euro ‘96, which can be viewed as mediated patriot games where nation is pitted against nation and matches are framed as contests between ‘us’ and ‘them,’ may actually strengthen dominant their national identities.

Poulton and Maguire (2012) concentrate on the dynamic interplay between national identity and media-sport during the London 2012 Summer Olympics. In particular, against the backdrop of current British identity politics, focus is placed on how members of “Team GB” were constructed and portrayed in the English press in the lead-up to and during London 2012. The dominant emotions of the larger geopolitical environment can be reflected in sporting passions, which frequently serve to reinforce or even create these social currents. The narrative of “plastic Brits” was (re)constructed and represented through texts and images in the English press during London 2012, and this is examined using a qualitative content analysis. This interpretive analysis was conducted on a cross-section of six daily English newspapers between August 3, 2012, the day of the opening ceremony, and August 19, 2012, 1 week following the closing ceremony. They present the three primary conclusions that arose from our examination of the press coverage: Although prevalent prior to the Olympic Games, the “plastic Brit” narrative was mostly absent during the Games; performances by “plastic” members of “Team GB” were de-emphasized in secret discourses about established-outsider relations: “plastic Brit” successes were praised, albeit less so than those of “true Brits,” while “plastic Brit” failures were largely pushed to the sidelines; hosting the Games and presenting the nation to the world encouraged journalists and politicians to (re)interpret and try to make sense of modern Britain.

Al-Atrash et al. (2016) determine the degree of group cohesion among Palestinian university team sports players and the variations in group cohesion based on university and team sport variables. 120 players made up the study’s sample. Group cohesion was assessed using the Group Environment Questionnaire (GEQ; Carron et al., 1985). The 34 items that make up the GEQ are divided into four domains. The findings showed that team sports players at Palestinian universities had a high degree of group cohesion, with 76.4% of the total score falling into this category. The GEQ domains were ranked as follows: group integration-social (83.2%), group integration task (77.8%), individual attraction to the group-task (74.8%), and individual attraction to the group-social (69.8%). Additionally, the results revealed statistically significant differences in the group integration-social domain based on the university variable. In light of the results, the researchers suggested that the university team formation concentrate on group cohesion.

Sheng and Gao (2019) conducted on foreign players in the Chinese Basketball League indicated a significant improvement in the overall technical level of the league. This improvement was attributed to the higher skill, physicality, strategy, and psychological attributes of foreign players compared to Chinese players, due to their extensive experience in various leagues such as the American, European, and Asian basketball leagues.

The presence of foreign players enhanced the competitive level of Palestinian players and strengthened the competitive power among the clubs in the Palestinian league. The Palestinian league is a relatively young league that has introduced foreign players into its teams. When discussing the presence of foreign players in the Palestinian Basketball League, it is essential to highlight the importance of collective teamwork and maintaining the cohesion of all team members to achieve victory. Cohesion is a trait that every coach strives for to ensure stable relationships among team members, positively impacting results.

According to Erikstad et al. (2018) cohesion is a fundamental element in sports performance, reflected in each team member’s commitment to the team’s goals and understanding of their required duties. Edmondson and Harvey (2017) also emphasize that cohesion encompasses two main dimensions: task cohesion, which refers to the degree of organization among group members and their commitment to achieving goals, and social cohesion, which relates to the group’s attractiveness and the aspects that create a collective appeal to outsiders (Filho et al., 2015; Yousef et al., 2019).

In this sense, cohesion can be defined as a dynamic process reflected in individuals’ interactions with the group, their attraction to it, and their tendency to unite and stay together to meet the group’s needs and shared goals (Carron and Brawley, 2012; Franz, 2012; Khomutova, 2015; Sung et al., 2008; Eys et al., 2019).

By establishing two equivalent random groups appropriate for the study’s purpose and choosing a sample of 20 junior basketball players between the ages of 13 and 15 (mean 15.1 SD 0.64), Yousef et al. (2020) use empirical research to investigate the effects of a recommended training regimen regarding several kinematic characteristics and jump shot (from the left and right side) for basketball beginners. Each of the two groups the experimental group and the control group—had 10 members. The experimental group received the recommended training program, while the control group received training using the traditional approach. The study’s findings showed that the new training regimen improved the values of a number of kinematic variables, including ball release height, angle, and velocity. The vertical velocity, one of the most improved variables in basketball jump shots, was particularly noteworthy. Additionally, this study suggests that the new jump shot training program be implemented for Palestinian school teams and junior clubs. It also highlights the significance of focusing on the kinematic variables influencing basketball juniors’ jump shot skills during training and the development of adolescents’ physical abilities.

Sajid et al. (2020) investigate whether (a) team efficacy and group cohesion improve volleyball players’ performance and (b) there is a correlation between volleyball players’ performance, team efficacy, and group cohesion. 200 female volleyball players competing at the school level answered questions regarding performance and cohesion efficacy. Two composite measures were used to examine the individual performance. The two-facilitating relationship between group cohesion, team efficacy, and athletic performance was validated by regression analysis. The results of statistical analysis indicated a positive correlation between team efficacy and group cohesion. Additionally, the findings demonstrated that while group cohesion has a negative impact on the performance of female volleyball players, team efficacy variables have a positive and significant impact on school volleyball players’ performance. In light of the results, it is recommended that school-level female volleyball players possess team efficacy in addition to other elements in order to make up for their shortcomings and accomplish their objectives. Team efficacy can raise the likelihood of enhancing team members’ compassion, trust, and amiable atmosphere through skill-related training, which could help the team accomplish its objectives more successfully. Both on and off the volleyball court, team members and their coaches should cultivate strong teamwork, helpful behaviors, and decision-making abilities in order to establish connections with group cohesion and team effectiveness.

Team cohesion can be observed in team sports, especially basketball, through synergy, cooperation, communication, and collective teamwork among the players themselves and between the coach and the players (Aliberti, 2022; Song and Zhang, 2021; Lu et al., 2021). Researchers believe that no team can execute offensive or defensive plans without quick analysis and understanding of game situations, effective communication, and strong cohesion between players and the coach.

Despite the importance of cohesion, Trull et al. (2023) noted that conflicts might arise among players, affecting team dynamics, such as competition for the same position. Filho and Basevitch (2021) pointed out that tensions could develop between new and old players within the team, with new players possibly experiencing anxiety about their position, role, and importance in the team (Levi and Askay, 2020).

Through their review of previous studies on team cohesion in the Palestinian environment, the researchers found that most studies only examine the relationship between the level and impact of team cohesion and certain psychological and social variables among local and foreign players in Palestine, such as the studies by Yousef et al. (2020), Al-Atrash et al. (2016) and Sajid et al. (2020). However, the topic of team cohesion between local and foreign players has not been addressed, despite the presence of foreign players in the Palestinian Basketball League.

By observing and following Palestinian basketball, the researchers noticed differences in the technical, strategic, physical, and psychological levels between foreign and local players. Reviews of player performance analyses, disciplinary records, personal fouls, scoring, and match records from the 2023 Palestinian Basketball League for teams with foreign players highlighted each player’s efficiency and contribution to their team’s success, as well as numerous issues and poor relationships between foreign and local players.

Discussions with coaches revealed that tactical plans often revolve around foreign players, emphasizing individual performance over cohesive team dynamics. This approach may underlie the instability and lack of unity among players. To address this issue scientifically, the researchers developed a specialized questionnaire to explore the impact of foreign players’ participation on team cohesion in the Palestinian Basketball League from the perspective of local players. The study aims to identify practical solutions to enhance cohesion and achieve the research objectives.

This study fills a notable gap in the literature by creating and developing a novel tool designed to assess cohesion within multicultural sports environments, particularly in leagues integrating foreign and local players. By offering a scientifically grounded measure, it enables researchers to analyze team dynamics more accurately and provides actionable recommendations for fostering better integration and cooperation among diverse player groups, thereby contributing to a more stable and cohesive team framework.

Discussions with coaches revealed that they often base their tactical plans on the foreign player, focusing on individual performance rather than fulfilling roles within the team framework. This approach may be the primary reason for the lack of stability and cohesion among players. Therefore, the researchers decided to study this problem scientifically by preparing a special questionnaire to understand the impact of foreign players’ participation in the Palestinian Basketball League on team cohesion from the perspective of local players, to contribute to finding solutions to achieve the study’s objectives.

### Research objectives

The following specific objectives were achieved:

*RO1.* This study attempts to evaluate, from the viewpoint of local players, the effects of foreign players’ involvement in the Palestinian Basketball League on team cohesion.

*RO2.* Verifying the variations in the study sample’s average answers concerning the effects of foreign players’ participation in the Palestinian Basketball League on team cohesion from the perspective of local players based on player status, years of experience, and player status?

### Research questions

The current study aimed to answer the following questions:

*RQ1.* What is the impact of foreign players’ participation in the Palestinian Basketball League on team cohesion from the perspective of local players?

*RQ2.* Do the average responses of the study sample regarding the impact of foreign players’ participation in the Palestinian Basketball League on team cohesion from the perspective of local players differ according to the variables of player status, years of experience, and player position?

Understanding the surroundings and circumstances in which the team plays is comparable to gaining insight into the effects of international players’ involvement in regional and national competitions. This is a crucial matter not only for the owner and coaches but also for every athletic director. As a result, this study contributes to the pertinent body of literature by enlarging the field of study and shedding light on the degree to which international players’ participation in the Palestinian basketball league affects both national coaches and Palestinian basketball players.

This paper is unique in that it examines the impact of foreign players’ participation in the Palestinian Basketball League on team cohesion from the perspective of local players. Most previous research has concentrated on the overall performance of teams and the influence of the playing environment, rather than examining the role of high-efficiency foreign players in team cohesion from the perspective of local players who may also be high-efficiency players for their respective national teams.

The remainder of this paper is organized as follows. Section 2 covers the previous literature review. Section 3 presents the research methodology and sample. The empirical findings and discussion are presented in section 4. Finally, section 5 summarizes the conclusion, policy implications, recommendations, and directions for future research.

## Literature review

Literature Review to provide a solid scientific foundation for the study, the theoretical framework relies on tools such as the Group Environment Questionnaire (Carron et al., 1985), which is considered a standard framework for understanding the dimensions of team cohesion. This framework enables a comprehensive exploration of how social, psychological, and cultural factors influence cohesion. Through this theoretical lens, the study aims to examine how foreign players can either enhance or hinder cohesion within local sports teams. In addition to its theoretical contributions, the study provides practical value by offering guidelines for sports clubs on selecting foreign players in a way that aligns with enhancing team cohesion, ultimately improving overall team performance. As emphasized by Schinke et al. (2021), research that bridges theoretical understanding and practical application directly contributes to better coaching and team management practices.

By reviewing previous studies, we find that many studies have highlighted and focused on the importance and role of foreign players in team cohesion. For instance, the study by Varmus et al. (2020) indicated that teams with a higher proportion of foreign players tend to perform better and achieve significant victories in tournaments. There is a substantial positive impact of foreign players on team morale, cohesion, and the overall dynamics and performance of the team.

Rana and Choudhary (2024) confirmed the significant influence of foreign players on the success of the team. It highlighted how local players gain exposure and experience, which enhances the overall performance of the team and raises the level of competition. Dixit (2023) emphasized the importance of having foreign players in the Indian league due to their substantial role in attracting a global audience to follow the league. Fans from around the world watch their favorite players compete, and the presence of foreign players from different countries and cultures helps break down barriers, enhance mutual understanding between different communities, and boost the economy and marketing in countries and leagues with a high presence of foreign players (Panagiotis et al., 2014; Chin, 2015; Haddera, 2015; Brisimis et al., 2018; Sullivan et al., 2023).

Ke et al. (2024) offer a cohesive framework for creating basketball team rosters that combines supervised and unsupervised machine learning models. In the unsupervised phase, they employed a clustering technique to classify the features that described player performance after reducing the number of features using principal components analysis (PCA). To create a sports team model that provides the best player type combination, we employed a basic neural network during the supervised learning stage. They combined two weighting techniques to create a rating system for players that took into account both their performance trends and their capacity for high-pressure play. They use the same framework for both the Women’s National Basketball Association (WNBA) and the NBA. Based on the clustering, team model, rates, and player salaries, the unified framework produced the ideal team roster. Their NBA framework found 10 distinct player clusters, four of which were categorized as elite clusters. Two players from two elite clusters and the top players from the other two non-elite clusters make up the team model. They employed various weighting techniques in their framework to assess players’ performance. They eventually applied the framework to the WNBA after discovering a strong correlation between the outcomes and actual facts of prior NBA games.

Duran (2023), conducted on basketball players across various levels, revealed differences in team cohesion levels among basketball players in Turkish leagues based on variables such as years of experience. Players with 1–7 years of experience showed higher levels of cohesion compared to more experienced players, who focused more on their performance and thoughtless about the team as a whole. The study also found that the higher the league level, the lower the cohesion, and the longer the period players spent with their teammates, the lower the overall team cohesion.

Despite the studies that highlighted the positive aspects of having foreign players in teams, there are also some studies showing the downsides. For example, the study by Paulauskas et al. (2024) revealed that the dominance of foreign players affects the technical and psychological maturity of Chinese players and the sustainable development of Chinese basketball to some extent. Similarly, Berri et al. (2022) showed that excessive reliance on foreign players has led to a significant reduction in playing time for Chinese players, resulting in a lack of experience and competition exposure.

Huang et al. (2022) showed that during all matches of the first phase of the CBA 2021–2022 season, Chinese players gained opportunities to prove themselves, such as playing longer than usual. This time was sufficient to explore the opportunities and challenges faced by Chinese players. The results indicated that in matches played in a closed tournament system without foreign players, Chinese players gained better opportunities, such as more playing time and shooting attempts since local starters and juniors also had more opportunities to prove themselves.

Dorak and Vurgun (2006) analyzed the relationship between empathy and team cohesion in terms of team sports, found that team cohesion levels decrease with increasing experience. The result related to league level was different, showing that cohesion increases with higher league levels, contrary to Tatar (2009), who indicated that playing in higher-tier tournaments reduces team cohesion as experienced players focus more on their performance rather than team unity. This was confirmed by Dorak and Vurgun (2006), who found that team cohesion decreases with increasing experience. Polat (2019) found that the longer the players played for their team, the lower their team cohesion in hockey (curling). Freire et al. (2022) indicated that task orientation appears to be a positive indicator of team cohesion, while self-centered orientation (ego) may positively predict group conflict and negatively affect task cohesion.

According to Risser et al. (2018) study on the main leagues in the US, clubs have a better record at home than away from home, and their home win percentage is over 50% (Courneya and Carron, 1992). In addition, the team’s track record and performance are important aspects of their home business.

Godfrey et al. (2021) investigated ethnic identity as a moderator of the relationship between ethnic diversity and young athletes’ perceptions of cohesion in interdependent sport teams (such as football). Ethnic diversity was found to be a negative predictor of both task and social dimensions of cohesion, according to a multilevel analysis of data from 272 young athletes nested within 24 teams. However, ethnic identity did not show up as a moderating factor. These results differ from those of recent studies that looked at comparable relationships in intercollegiate settings. This demonstrates the intricacy and significance of investigating how ethnic diversity affects team functioning-oriented variables at various athletic competition levels. Their research sheds light on the impact of ethnic diversity in youth sports and highlights important developmental processes (like identity formation) that researchers should consider in future studies, even though replication studies are required to fully comprehend the validity of the current findings.

Guimarãe et al. (2018), which differentiated between foreign and local players in the Portuguese basketball league, showed that foreign players outperformed local players in almost all offensive and defensive tasks of the game. The idea of team success, expressed through the joint contribution of local and foreign players, reflects the principles behind the employment of basketball players in their positions on the court and the philosophy of play adopted by the coaches.

Athanasios et al. (2016) indicated that among the many factors that can positively or negatively affect the outcome of a basketball game and crucially contribute to achieving goals is cohesion among its members. Sports teams characterized by high cohesion tend to cooperate better, communicate positively, and perform more effectively. A review of related literature noted that most studies focused on factors affecting team cohesion, with homogeneity among players being the most influential element according to coaches, while financial factors were considered the least important.

Meletakos et al. (2016) analyze the level of competition in Greek handball and basketball championships with the quantity and presence of international players. For basketball, the studied periods are 1965–1966 to 2012–2013 (*n* = 47) and 1983–1984 to 2012–2013 (*n* = 30) for handball. In handball, foreign players first debuted in 1999, although in basketball, they first appeared in 1988. Findings indicated that the macroeconomic standing of the nation influences the number of foreign players per team, which raises both overall and relegation-level competitiveness. Compared to basketball, handball had a later and smaller influx of international players.

Muthiane and Rintaugu (2015) conducted on basketball players in the Kenyan league, indicated that players disliked other players in their teams due to laziness, pride, and selfishness. However, 96% of players celebrated victories as a team, and 76% lost as a team. Teams with high levels of cohesion won more matches compared to teams that lost more matches. The study also found no differences between males and females in team cohesion, concluding that team size affects cohesion, with smaller teams enjoying higher cohesion than larger teams.

Olushola et al. (2013) identify the elements of school-based athletic programs that benefit African-American girls in the long run. The program was implemented at a high school basketball program that was founded on positive youth development. Semi-structured interviews with program stakeholders and current and former players were used to gather data. The program’s success is based on four fundamental values: civic engagement, discipline, education, and family. The findings show that consistent mission and execution, as well as flexible—rather than standard—design commitment to program values, are necessary for successful programs. Implications for the planning and execution of sports programs aimed at enhancing African-American girls’ academic and athletic achievement are examined.

Developing high-identity fans by the club’s outstanding play and assisting the team in maintaining a stable and long-term presence in the home city are two examples of localization values (Kuo et al., 2012). The Formosa Dreamers’ 2017–2018 season record has not yet matched 50% of the home victory percentage that academics had predicted. Even though the team’s percentage of home victories has increased in the new season, they still need to evaluate this aspect of the team thoroughly, evaluate and strengthen the current roster, and replace and strengthen players as needed in order to keep improving the strength of the game. For a basketball team, a yearly training program is necessary and has to be well-planned. The thorough training plan might contain environmental adaptation in addition to considering the level of instruction and the expertise and high productivity of instructors in this sector.

From the above, cohesion can be defined as a dynamic process with several different dimensions and indicators that can indicate its strengths or weaknesses. It reflects the group’s tendency to unite and stay united in the pursuit of meeting its members’ needs and achieving the group’s common goals (Carron and Brawley, 2012; Shor and Galily, 2012; Laios and Alexopoulos, 2014; Eys et al., 2019; Huang et al., 2022). Group cohesion can be identified as a fundamental element for sports performance, from the perspective of task cohesion or social cohesion (Do Nascimento Junior et al., 2019). Task cohesion refers to the desire of group members to work together to achieve a common goal, while social cohesion refers to the degree of satisfaction of members’ emotional needs (Eys et al., 2019). Despite this, it is also emphasized that there is a negative group process related to conflict, defined by disagreements and personal issues, and viewed through the lens of the negative social dimension, task performance, and relationship conflicts (Heuzé et al., 2006; Jones, 2015).

Lencioni (2005) found that selfish players do not trust others, viewing them as unequal players, which jeopardizes team goals since some foreign players consider themselves better than their teammates. According to Liu et al. (2002), one of the key elements affecting contemporary players’ performance is their familiarity with domestic and international venues and facilities. The facilities and fields that athletes are familiar with both on and off the field range greatly. Furthermore, kids might exhibit greater self-confidence and their physical and mental states are more stable when they play at home. The location will also have an impact on athletes’ technical performance (Ding et al., 2019). According to Matsuoka et al. (2003), the team’s performance and record have an impact on the supporters’ willingness to continue supporting the team and return to the game.

By broadening the scope of the investigation and emphasizing the degree to which foreign players’ involvement in the Palestinian Basketball League influences both national coaches and Palestinian basketball players, this study seeks to fill the research gap that currently exists in the pertinent literature.

Existing standard tools for measuring team cohesion, such as the Group Environment Questionnaire (Carron et al., 1985; Jones, 2015), were primarily designed for specific cultural and sports contexts, often in Western settings. However, these tools fail to account for the cultural and social nuances of non-Western environments, such as the Palestinian context. This limitation underscores the importance of developing new, context-sensitive tools. As noted by Schinke et al. (2021), creating such tools must be grounded in strong scientific justification, particularly when current measures are inadequate for capturing phenomena in specific cultural settings.

By designing and developing a new tool to evaluate cohesion in multicultural sports environments, especially in leagues that integrate local and foreign players, this study closes a significant gap in the literature. By providing a measure with scientific backing, it helps researchers better understand team dynamics and offers practical suggestions for improving cooperation and integration between various player groups, which helps create a more stable and cohesive team structure.

## Design and methodology

### Research design

The study aimed to identify the impact of the participation of foreign players in the Palestinian Basketball League on team cohesion from the perspective of local players. It also sought to determine the differences in team cohesion among local players based on variables such as player status, years of experience, and player position.

To answer the study’s questions, a quantitative design (survey questionnaire) was used to collect data from the study sample, relying on literature and previous studies related to the topic, such as the studies by Carron et al. (1985).

The researchers used the descriptive analytical method to determine the arithmetic means, standard deviations, percentages, frequencies, one-way ANOVA, LSD test, and independent t-test to identify the differences in the impact of foreign players’ participation on team cohesion according to the variables of playing experience, player status, and player position. The Pearson Correlation Coefficient was used to verify the internal consistency validity of the scale, and Cronbach’s Alpha was used to ensure the reliability of the study tool.

### Reasons for developing a new measure

Existing standard tools for measuring team cohesion, such as the *Group Environment Questionnaire* (Carron et al., 1985), were primarily designed for specific cultural and sports contexts, often in Western settings. However, these tools fail to account for the cultural and social nuances of non-Western environments, such as the Palestinian context. As noted by Schinke et al. (2021), developing new tools must be supported by strong scientific justification, such as the inadequacy of current measures to assess phenomena in specific contexts.

### Features of the new measure

The newly developed measure in this study considers the cultural and social specificities of the Palestinian context. It is designed to capture three main dimensions:

1. Psychological Dimension: Focuses on players’ feelings toward the team and mutual psychological support.
2. Social Dimension: Reflects the relationships among team members and their level of integration.
3. Team Belonging Dimension: Measures the players’ sense of belonging and loyalty to their team.

### Validity verification

As emphasized by Schinke et al. (2021), validating a new tool is crucial to ensure its credibility and effectiveness. In this study, several validation methods were employed:

1. Construct Validity: Factor analysis was used to confirm the alignment of the tool’s structure with the measured dimensions.
2. Criterion Validity: The new tool’s results were compared with those of established cohesion measurement tools.
3. Reliability: Cronbach’s alpha was measured, and the tool demonstrated a high reliability score of 0.97.

### Participants

The study was conducted in 2023 during the Premier Basketball League championship in Palestine. An electronic questionnaire was prepared and sent to all local Palestinian players participating in the league, totaling 121, through coaches and administrators in clubs and the Palestinian Basketball Federation. A sample of 84 basketball players responded to the questionnaire, aged between 18 and 38 years. The sample represents approximately 68% of the population, as shown in Table 1, which presents the characteristics of the study sample participants.

**Table 1:** Study sample characteristics.

Independent variables	Variable level	Number	Percentage (%)
Player’s experience	5 years or less	49	58.3
	From 6 _ 10 years	24	28.6
	10 years and more	11	13.1
	Total	84	100%
Player’s attribute	Starter	30	35.7
	Substitute	54	64.3
	Total	84	100%
Player’s position	Guard	44	52.4
	Forward	26	31
	Center	14	16.7
	Total	84	100%

Source: Authors’ own work.

### Study instrument

In light of the study objectives, the researchers designed a tool (questionnaire) to collect the necessary data from the study sample after reviewing the study literature and previous related studies, such as the study by Carron et al. (1985). Items were adapted from Carron et al. (1985) group environment questionnaire and validated by three sports psychology experts. The final version of the tool consisted of 30 items distributed across three dimensions: social dimension, psychological dimension, and team belonging dimension. The response weights related to the cohesion scale items were distributed according to the five-point Likert scale with five responses: (strongly agree, agree, moderately agree, slightly agree, and strongly disagree). Thus, the highest response score is 5, and the lowest response score is:

### Scientific characteristics of the study instrument

#### Validity of the study instrument

The researchers used content validity in the first step and internal consistency validity in the second step after distributing the scale to a pilot sample of 10 basketball players, who were excluded from the original study sample. Pearson correlation coefficients between the fields and the overall score for each scale were then calculated, as shown in Tables 2, 3. The details are as follows:

**Table 2:** Internal consistency validity.

Number	Social dimension	Value(r)	Significance level
1	Our team players have become more cooperative in developing their skills with the presence of foreign players.	0.784**0	0.000
2	The professional player helps solve problems that occur during training.	0.875**0	0.000
3	The professional player contributes to feedback for any participation with team members.	0.846**0	0.000
4	Foreign players, along with the rest of the team members, take responsibility for the competition results regardless of the outcome	0.822**0	0.000
5	Foreign players share their viewpoints with team members to set team goals.	0.781**0	0.000
6	Foreign players cooperate with the rest of the team members to achieve satisfactory results.	0.867**0	0.000
7	Foreign players participate with team members in social issues.	0.718**0	0.000
8	Team members exchange encouraging phrases with foreign players for better performance.	0.623**0	0.000
9	Foreign players can play a significant role in helping their teams overcome challenges in competitions	0.723**0	0.000
10	Foreign players participate in various social events with team members.	0.792**0	0.000
Number	Psychological dimension	Value(r)	Significance level
1	My confidence increases when foreign players are in the lineup.	0.798**0	0.000
2	The team’s performance is convincing when foreign players play.	0.858**0	0.000
3	Foreign players show responsibility when they fail.	0.844**0	0.000
4	Foreign players do not care about the team’s results regardless of the outcome.	0.766**0	0.000
5	Foreign players care about the integrated effort of the team.	0.831**0	0.000
6	Foreign players show leadership traits on the field.	0.825**0	0.000
7	Local players harmonize with foreign players during competition.	0.684**0	0.000
8	Foreign players interact with the team captain’s instructions.	0.826**0	0.000
9	Foreign players respond efficiently to game changes.	0.753**0	0.000
10	Foreign players celebrate with team members when goals are scored.	0.759**0	0.000
Number	Team affiliation dimension	Value(r)	Significance level
1	Foreign players do not show interest in moving to another team	0.764**0	0.000
2	Foreign players share the team’s interests with other team members.	0.687**0	0.000
3	The foreign players complain about the club’s management.	0.646**0	0.000
4	Foreign players follow the results of teams in the Palestinian basketball league	0.788**0	0.000
5	When losing, the foreign players show sadness.	0.876**0	0.000
6	The foreign players exert all their efforts to achieve victory	0.670**0	0.000
7	Foreign players are interested in showcasing themselves to the fans.	0.749**0	0.000
8	Foreign players show respect for the club.	0.738**0	0.000
9	Foreign players interact with the team’s fans.	0.859**0	0.000
10	Foreign players show interest in the team’s history and achievements.	0.867**0	0.000

**There is a statistically significant relationship at 𝛼 ≥ 0.01. Source: Authors’ own work.Bold values indicate statistically significant correlations at α ≤ 0.01, meaning there is a meaningful relationship between the related variables.

**Table 3:** Internal consistency validity of the scale dimensions.

No.	Dimensions	Value(r)	Significance level
1	Social dimension	0.949**	0.000
2	Psychological dimension	0.927**	0.000
3	Team affiliation dimension	0.953**	0.000

**A Statistically Significant Relationship at 𝛼 ≥ 0.01. Source: Authors’ own work.

#### Content validity

The researchers presented the cohesion scale to a group of experts and specialists to provide their opinions on the wording, clarity, and quality of representation of the items for the relevant fields and the subject matter they represent in each scale. After considering the experts’ opinions and making the required adjustments, the final version of the tool consisted of 30 items distributed across three dimensions, thus achieving the intended purpose of the tool.

#### Internal consistency validity

The results of Table 2 indicate that there is a statistically significant relationship at the significance level *α*( ≤ 0.01) between all domains and the total score of the coherence scale. The Pearson correlation coefficient values ranged between (0.623 and 0.876), which means that the scale is valid and achieves the purpose of its use.

The results of Table 3 indicate that there is a statistically significant relationship at the significance level (α ≤ 0.01). between all dimensions and the total score of the coherence scale. The Pearson correlation coefficient values ranged between (0.927–0.953), which means that the scale is valid and achieves the purpose of its use.

#### The reliability of the study tool

To verify the reliability of the scale, Cronbach’s Alpha formula was used for the same exploratory sample, and the results of Table 4 demonstrate this.

**Table 4:** Reliability coefficients of the coherence scale.

No.	Dimensions	Number of items	Cronbach’s alpha
1	Social dimension	10	0.93
2	Psychological dimension	10	0.93
3	Team affiliation dimension	10	0.92
	Overall scale	30	0.97

Source: Authors’ own work.

The results of Table 4 indicate that the overall reliability coefficient of the coherence scale was 0.97, and the reliability coefficients for the dimensions ranged between 0.92 and 0.93. This means that the scale has a high degree of reliability and is suitable for achieving the study’s purposes.

### Data collection procedures

The researchers designed the survey form using Google Forms to collect data from the study sample. They sent the questionnaire link to all local players participating in the Palestinian Basketball League through direct contact with players, administrators, and the Basketball Federation, as well as through social media (WhatsApp, Facebook, Telegram).

The questionnaire included a box indicating that participants had the right not to respond and that participation in the study was voluntary. All information obtained would be used solely for research purposes. The data collection process from the study sample took 25 days during the year 2023.

### Data analysis

The researchers obtained the data through the participants’ responses to the questionnaire prepared using Google Drive, converting the responses into an Excel sheet. They then converted and coded the variables into quantitative numbers and transferred them to the Statistical Package for the Social Sciences (SPSS) for analysis.

The researchers used the descriptive analytical method to determine the means, standard deviations, percentages, and frequencies. They used the One-way ANOVA and LSD test to determine if there were statistically significant differences according to the variables of experience and player position and to identify the nature of differences between the levels of experience and player position. The Independent t-test was used to determine differences in the impact of foreign player participation on team coherence, according to the player characteristic variable. The Pearson Correlation Coefficient was used to confirm the internal consistency validity of the scale, and Cronbach’s Alpha formula was used to confirm the reliability coefficients of the study tool.

## Empirical findings and discussion

### Results of the first question

What is the impact of the participation of foreign players in the Palestinian Basketball League on team coherence from the perspective of local players?

To answer this question, the mean and standard deviation for each item and its respective dimension, as well as the overall score for team coherence, were calculated. To interpret the results, the following relative weights were used: 80% and above: The impact of foreign player participation in the Palestinian Basketball League is very high. 70–79.99%: The impact of foreign player participation in the Palestinian Basketball League is high. 60–69.99%: The impact of foreign player participation in the Palestinian Basketball League is moderate. 50–59.99%: The impact of foreign player participation in the Palestinian Basketball League is low. Below 50%: The impact of foreign player participation in the Palestinian Basketball League is very low. The results of Table 5 illustrate this.

**Table 5:** Means, standard deviations, and scores for the items of the coherence scale among foreign players in the Palestinian basketball league in Palestine (*N* = 84).

No.	Social dimension items	Mean	Standard deviation	Score (%)
7	Foreign players participate in team members’ social issues.	4.00	1.60	Very High
8	Team members and foreign players exchange encouraging phrases.	3.95	1.31	High
1	Our team members became more cooperative in developing skills with foreign players.	3.51	1.52	High
3	The professional player contributes feedback to team members.	3.44	1.74	Moderate
9	Foreign players help team members find solutions to challenges.	3.26	1.61	Moderate
10	Foreign players participate in different social events with team members.	3.17	1.66	Moderate
5	Foreign players share views with team members to set team goals.	3.11	1.65	Moderate
4	Foreign players take responsibility with the team for competition results.	2.85	1.78	Low
6	Foreign players collaborate with team members to achieve satisfactory results.	2.70	1.70	Low
2	Foreign players share the team’s interests with members.	2.57	1.83	Low
	Overall Social Dimension	3.26	1.29	Moderate
No.	Psychological dimension items	Mean	Standard deviation	Score
11	My self-confidence increases when foreign players are in the lineup.	3.44	1.42	Moderate
15	Foreign players care about the team’s integrated effort.	3.36	1.41	Moderate
16	Foreign players show leadership traits on the field.	3.36	1.33	Moderate
19	Foreign players respond efficiently to game changes.	3.31	1.50	Moderate
17	Local players blend well with foreign players during competition.	3.30	1.62	Moderate
14	Foreign players do not care about the team’s results.	3.23	1.59	Moderate
12	The team’s performance is convincing when foreign players play.	3.21	1.35	Moderate
18	Foreign players respond to the team captain’s instructions.	3.08	1.58	Moderate
20	Foreign players celebrate with team members when goals are scored.	3.05	1.69	Moderate
13	Foreign players show responsibility when failing.	2.81	1.61	Low
	Overall Psychological Dimension	3.21	1.20	Moderate
No.	Team affiliation dimension items	Mean	Standard deviation	Score
27	Foreign players care about showing themselves to the fans.	3.83	1.41	High
23	Foreign players complain about club management.	3.80	1.25	High
28	Foreign players show respect for the club.	3.57	1.24	High
22	Foreign players share the team’s interests with team members.	3.52	1.47	High
21	Foreign players do not care about transferring to another team.	3.46	1.56	Moderate
24	Foreign players follow the results of teams in the Palestinian Basketball League.	3.44	1.31	Moderate
29	Foreign players interact with team fans.	3.40	1.38	Moderate
30	Foreign players show interest in the team’s history and achievements.	3.37	1.45	Moderate
26	Foreign players put all their efforts into achieving victory.	3.36	1.40	Moderate
25	Foreign players show sadness when losing.	3.21	1.47	Moderate
	Overall Team Affiliation Dimension	3.50	1.07	High

Source: Authors’ own work.

The highest response is (5) points. The results of Table 5 indicate that the overall score for the social dimension among players of the Premier League basketball clubs in Palestine was moderate, with an average response of (3.26). The score was very high for item (7), with an average response of (4.00), while item (2) had a low score, with an average response of (2.57). Similarly, the overall score for the psychological dimension among players of the Premier League basketball clubs in Palestine was moderate, with an average response of (3.21). All items in this dimension had moderate scores except for item (13), which had a low score of (2.81). The overall score for the team affiliation dimension among players of the Premier League basketball clubs in Palestine was high, with an average response of (3.50). Item (27) ranked first in this dimension with an average response of (3.83), while item (25) ranked last with an average response of (3.21).

#### Summary of the results of the first question

The results of Table 6 indicate that the impact of foreign player participation in the Palestinian Basketball League on team coherence from the perspective of local players was moderate, with an average response of (3.32). The scores for the dimensions ranged from moderate to high, with average responses between (3.21 and 3.50). The highest response was for the team affiliation dimension, which was high with an average score of (3.50), followed by the social dimension, which was moderate with an average score of (3.26). The lowest response was for the psychological dimension, which was moderate with an average score of (3.21).

**Table 6:** Means, standard deviations, and scores for the dimensions of the coherence scale among players of the premier league basketball clubs in Palestine (*N* = 84).

No.	Dimensions	Mean	Standard deviation	Score (%)	Rank
1	Team Affiliation	3.50	1.07	70	First
2	Social Dimension	3.26	1.29	65	Second
3	Psychological Dimension	3.21	1.20	64	Third
	Overall Coherence	3.32	1.12	66	Moderate

*The highest response score is (5) points. Source: Authors’ own work.

The results show that the overall score for the impact of foreign player participation in the Palestinian Basketball League on team coherence from the perspective of local players is moderate. The team affiliation dimension had the highest response, indicating a high impact. The researchers attribute this result to the fact that team affiliation is a top priority for players regardless of their status, age, or whether they are local or foreign players. Foreign players strive to integrate quickly with local players to be accepted by both the players and the fans. This is why the response for the team affiliation dimension, particularly item 27, which states “Foreign players care about showing themselves to the fans,” was high. This indicates that foreign players are keen to market themselves to the fans through their affiliation and loyalty to the team. Additionally, Panagiotis et al. (2014); Chin (2015); Brisimis et al. (2018); and Sullivan et al. (2023) highlighted the importance of foreign players in global marketing, while Dixit (2023) pointed out the contribution of foreign players to marketing and the economy in sports.

### The second question

What is the impact of foreign players’ participation in the Palestinian basketball league on team cohesion from the perspective of local players, according to variables (experience in playing, playing position, player trait)?

To identify the differences in the impact of foreign players’ participation in the Palestinian basketball league on team cohesion, one-way ANOVA was used, and the results from Tables 7–12 illustrate this. Additionally, an independent t-test was used for two independent groups to determine the differences in the impact of foreign players’ participation in the Palestinian basketball league on team cohesion according to the player trait variable, as shown in Table 6. Below is the presentation of the results according to the independent variables:

**Table 7:** Mean scores and standard deviations for the impact of foreign players’ participation in the Palestinian basketball league on team cohesion according to the experience in playing variable (*N* = 84).

Cohesion dimensions	5 years or less (*N* = 49)	6–10 years (*N* = 24)	More than 10 years (*N* = 11)
Social cohesion	3.56 (SD = 1.14)	2.35 (SD = 1.11)	3.89 (SD = 1.40)
Psychological cohesion	3.31 (SD = 1.23)	2.70 (SD = 0.86)	3.90 (SD = 1.27)
Team affiliation	3.47 (SD = 0.92)	2.81 (SD = 1.05)	3.90 (SD = 1.14)
Total Score	3.53 (SD = 1.02)	2.62 (SD = 0.93)	3.89 (SD = 1.24)

Source: Authors’ own work.

**Table 8:** One-way ANOVA results for the effect of foreign players’ participation in the Palestinian basketball league on team cohesion according to playing experience (*N* = 84).

Team cohesion dimensions	Source of variation	Sum of squares	Degrees of freedom (DF)	Mean square	*F* value	Significance level
Social Cohesion	Between Groups	28.82	2	14.41	10.62	0.001*
	Within Groups	109.91	81	1.357		
	Total	138.73	83			
Psychological Cohesion	Between Groups	11.99	2	5.995	4.551	0.013*
	Within Groups	106.71	81	1.317		
	Total	118.70	83			
Team Affiliation	Between Groups	18.81	2	7.904	8.084	0.001*
	Within Groups	79.19	81	0.978		
	Total	95.00	83			
Total Team Cohesion	Between Groups	17.71	2	8.855	8.326	0.000*
	Within Groups	86.14	81	1.064		
	Total	103.85	83			

Differences are statistically significant at the level (α ≤ 0.05). Source: Authors’ own work.

**Table 9:** Results of the L.S.D. test for pairwise comparisons between the mean scores for team cohesion and its dimensions significantly at the significance level among basketball players according to playing experience (*N* = 84).

Dimensions of team cohesion	Mean score	Playing experience categories
Social Cohesion	3.56	1.21*
2.35	−1.21*	−1.54*
3.89	0.33	1.54*
Psychological Cohesion	3.31	0.61*
2.70	−0.62*	−1.20*
3.90	0.42	1.20*
Team Affiliation	3.47	0.92*
2.81	−0.92*	−1.08*
3.90	0.15	1.08*
Overall Score	3.53	0.91*
2.62	−0.91*	−1.27*
3.89	0.35	1.27*

Differences are statistically significant at the significance level (α ≤ 0.05). Source: Authors’ own work.

**Table 10:** Mean and standard deviations for the impact of foreign players’ participation in the Palestinian basketball league on team cohesion according to the position variable (*n* = 84).

Team cohesion dimensions	Position	Number	Mean	Standard deviation
Social collective	Guard	44	3.22	1.32
	Forward	26	3.85	1.10
	Center	14	2.25	0.80
Psychological collective	Guard	44	3.05	1.19
	Forward	26	3.86	1.09
	Center	14	2.50	0.82
Team membership	Guard	44	3.43	1.11
	Forward	26	3.95	0.84
	Center	14	3.85	0.96
Overall cohesion	Guard	44	3.23	1.13
	Forward	26	3.88	0.94
	Center	14	2.53	0.79

Source: Authors’ own work.

**Table 11:** Results of one-way ANOVA for the significance of the impact of foreign players’ participation in the Palestinian basketball league on team cohesion according to the position variable (*n* = 84).

Team cohesion dimensions	Source of variation	The sum of squares deviation	Degrees of freedom	Mean square	F value	Significance level
Social Collective	Between Groups	23.63	2	11.818	8.318	0.001*
	Within Groups	115.09	81	1.421		
	Total	138.72	83			
Psychological Collective	Between Groups	19.09	2	9.547	7.764	0.001*
	Within Groups	99.60	81	1.230		
	Total	118.69	83			
Team Membership	Between Groups	11.35	2	5.679	5.499	0.006*
	Within Groups	83.64	81	1.033		
	Total	94.99	83			
Overall Social Cohesion	Between Groups	17.39	2	8.697	8.148	0.001*
	Within Groups	86.45	81	1.067		
	Total	103.84	83			

*Statistically significant differences at the significance level (α ≤ 0.05). Source: Authors’ own work.

**Table 12:** Results of the LSD test for pairwise comparison of the mean scores for team cohesion and its statistically significant dimensions among basketball players according to the playing position variable (*n* = 84).

Team cohesion dimensions	Mean	Playing position categories
		Guard
Social Collective	3.22	
	3.85	0.63*
	2.25	−0.97*
Psychological Collective	3.05	
	3.86	0.80*
	2.50	−0.55
Team Membership	3.43	
	3.95	0.51*
	3.85	−0.58
Overall Cohesion	3.23	
	3.88	0.65*
	2.53	−0.70*

*Statistically significant differences at the significance level (α ≤ 0.05). Source: Authors’ own work.

#### Experience in playing

The results in Table 8 indicate statistically significant differences at the significance level (*α* ≤ 0.05) in the overall degree of team cohesion and its dimensions (social cohesion, psychological cohesion, team affiliation) in the effect of foreign players’ participation in the Palestinian basketball league. These differences are attributed to the variable of playing experience. To determine the sources of these differences, the Least Significant Difference (L.S.D) test was used for pairwise comparisons between the means, as shown in Table 9.

**Figure 1:**
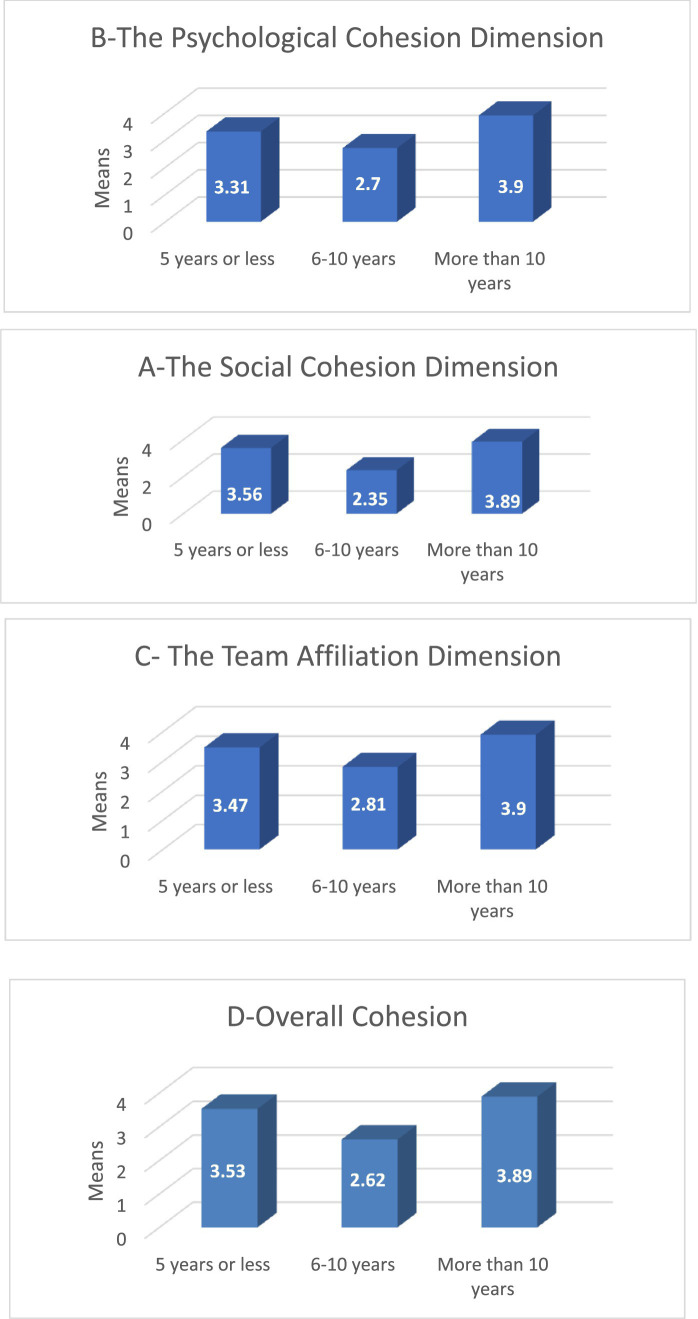
The average response to the dimensions of cohesion among local players in the Palestinian Basketball League according to the playing experience variable. Source: Authors’ own work.

**Figure 2:**
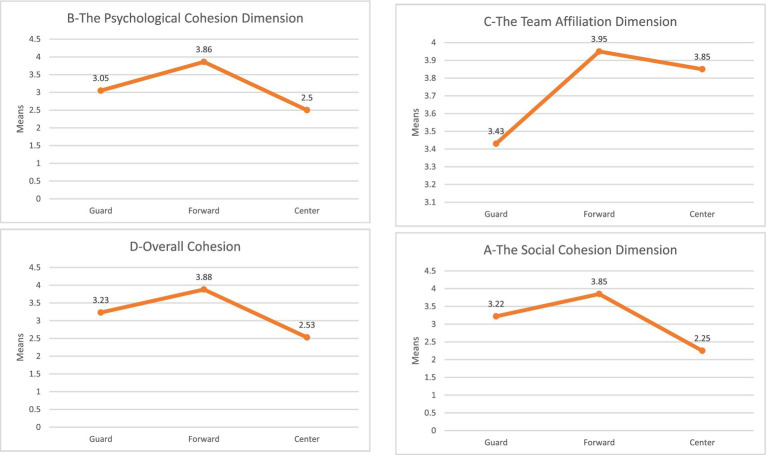
The average response to the dimensions of cohesion among local players in the Palestinian Basketball League according to the position variable. Source: Authors’ own work.

**Figure 3:**
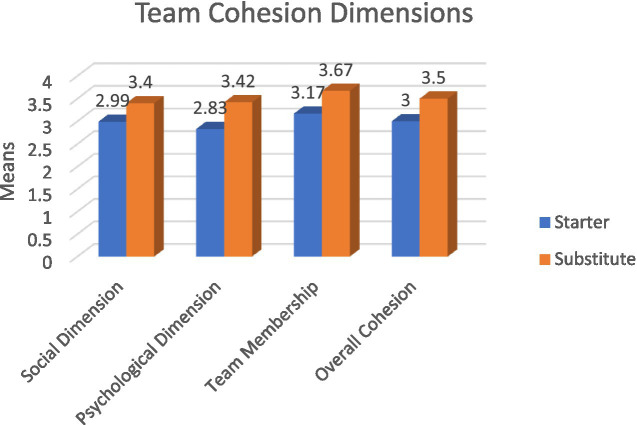
The average response to the dimensions of cohesion among local players in the Palestinian Basketball League according to the player status variable. Source: Authors’ own work.

The average response for the dimension of social cohesion in the impact of foreign players’ participation in the Palestinian basketball league is attributed to the variable of playing experience (*n* = 84). The results from Table 9 indicate statistically significant differences at the significance level (*α* ≤ 0.05) in the overall team cohesion and its dimensions (social dimension, psychological dimension, affiliation dimension) in the impact of foreign players’ participation in the Palestinian basketball league attributed to the variable of playing experience between (less than 5 years) and (6–10 years), favoring (less than 5 years), and between (more than 10 years) and (6–10 years), favoring (more than 10 years). However, there are no statistically significant differences between (less than 5 years) and (more than 10 years). Figures 1–3 illustrate this.

The researchers attribute these results to the perception of less experienced players that foreign players bring valuable expertise that can be leveraged and learned from, despite having fewer playing opportunities without the presence of foreign players. This aligns with Berri et al. (2022), who found that foreign players playing more than local Chinese players excel technically and physically and are capable of attracting crowds and excitement.

The researchers also suggest that more experienced local players with over 10 years of experience see foreign players as genuine supporters in improving team performance and increasing competitiveness. This is consistent with findings by Filho and Basevitch (2021) suggesting that both new and veteran players strive to compete in team dynamics and improve it. Additionally, the researchers believe that those with over 10 years of experience responded on the cohesion scale to their need for foreign players to develop the team, although this result contradicts the findings of Duran (2023) in the Turkish league and Dorak and Vurgun (2006), which suggest that cohesion decreases with experience.

#### Position variable

The results of Table 11 indicate statistically significant differences at the significance level (*α* ≤ 0.05) in the overall cohesion of the team and its dimensions (social collective, psychological collective, team membership) in the impact of foreign players’ participation in the Palestinian basketball league attributed to the position variable. To identify the sources of differences, the LSD test was used for pairwise comparison of means, and the results of Table 12 demonstrate this.

The results of Table 12 indicate statistically significant differences at the significance level (α ≤ 0.05) in the overall team cohesion and its dimensions (social collective, psychological collective, team membership) in the impact of foreign players’ participation in the Palestinian basketball league attributed to the playing position variable. Differences are found between (Guard position) and (Forward position) and (Center position) in favor of (Forward position) and between (Guard position) and (Center position) in favor of (Guard position).

The researchers attribute this result to the fact that the (Forward position) in the field has tasks that require more communication and interaction with all players compared to other positions. Forwards, whether Power Forward or Small Forward, are assigned multiple tasks between offense and defense and require continuous movement. Therefore, coaches need to select players with multiple skills and usually tall stature who can be effective under the basket both offensively and defensively. Selecting experienced players should positively affect the team’s dynamics and improve its performance (Filho and Basevitch, 2021).

#### Player status variable

The results of Table 13 indicate statistically significant differences at the significance level (*α* ≤ 0.05) in the overall team cohesion and its dimensions (psychological dimension, team membership) among players of the elite basketball clubs in Palestine attributed to the player status variable, in favor of the substitute players. However, there are no statistically significant differences in the social dimension based on the player status variable.

**Table 13:** Results of the t-test for the significance of differences in the impact of foreign players’ participation in the Palestinian basketball league on team cohesion according to the player status variable (*n* = 84).

Team cohesion dimensions	Player status	Number	Mean	Standard deviation	*t*-value	Significance level*
Social Dimension	Starter	30	2.99	1.27	−1.39	0.16
	Substitute	54	3.40	1.29		
Psychological Dimension	Starter	30	2.83	1.23	−2.22	0.03*
	Substitute	54	3.42	1.12		
Team Membership	Starter	30	3.17	1.08	−2.11	0.04*
	Substitute	54	3.67	1.02		
Overall Cohesion	Starter	30	3.00	1.14	−2.00	0.04*
	Substitute	54	3.50	1.07		

*Statistically significant differences at the significance level (α ≤ 0.05). Source: Authors’ own work.

The results showed no differences in the social dimension between substitute and starter players. The researchers attribute this result to the fact that the items designed in the social dimension scale equally account for social interactions and community events. Therefore, Palestinian basketball players, regardless of their status, find that foreign players socially integrate with the team because the Palestinian community fosters social cohesion and is not isolated. Filho et al. (2015) indicated that social cohesion creates a collective attraction for outsiders.

The differences in the team membership and psychological dimensions in favor of the substitute players are attributed by the researchers to the fact that although substitute players participate less than starter players, they have a sense of belonging and trust in foreign players, believing they can enhance team cohesion through their performance and spectator engagement. Starter players, on the other hand, may feel pressured to prove their abilities to the fans compared to foreign players. Özmen (2019) found that the shooting efficiency of foreign starter players was higher than that of local players.

## Conclusion and policy implications

### Conclusion

In light of the results obtained from the study, the researchers concluded the following key findings. The overall cohesion score was moderate, with the team membership dimension scoring the highest. The variable of playing experience showed differences between (less than 5 years) and (6–10 years) in favor of (less than 5 years), and between (more than 10 years) and (6–10 years) in favor of (more than 10 years).

The results related to playing position favored the Forward player, followed by the Guard player. The study found statistically significant differences at the level of (α ≤ 0.05) in the overall team cohesion and its dimensions (psychological dimension, team membership) among basketball players in Palestine in favor of substitute players. There were no statistically significant differences in the social dimension based on the player status variable.

Conversely, it appears that the Palestinian basketball team’s increased reliance on foreign players has affected the team’s level and performance index, which measures the competitiveness of the league’s top squad. This makes sense because the top clubs in both sports are often the ones with the resources to sign the greatest number of international players possibly the best players as well as the most financial resources. The findings of earlier research, which show that a team with talented foreign players performs better, are consistent with this. Furthermore, international players’ incorporation into the teams happened more organically because basketball was already a professional sport when they came, which enhanced the Palestinian team’s performance and competitiveness.

In summary, due to basketball’s higher professional status than football or handball, international players have emerged in Palestine later in the past 20 years and larger numbers. The macroeconomic conditions of the nation influence the entrance of international players, which in turn boosts participation and competitiveness. However, there has been a noticeable change in the performance of Palestinian players and their level of competition, particularly for lower-tier clubs, as the number of foreign players in the Palestinian Basketball League grew.

In basketball, the number of foreign players who have participated in leagues has increased, which has improved Palestinian players’ performance recently. It has also made the Palestinian National Basketball League more competitive because more foreign players have been evenly distributed among all teams, which has improved the caliber, output, and accomplishments of the top teams competing in competitions throughout Europe, Asia, and Africa. Furthermore, Palestinian teams have won several titles in the last two decades in the basketball competitions they play in outside of Palestine thanks to the participation of international players, both in terms of numbers and quality.

### Managerial implications and recommendations

Based on study’s findings, our results suggest some significant managerial implications: the researchers recommend the importance of having foreign players to enhance the cohesion level among players. It is important to select foreign players based on their dynamic attributes to maintain team stability and development. Emphasize the involvement of substitute players, who expressed the importance of cohesion and team membership with the presence of foreign players. It is necessary to prepare the team psychologically and socially to accept and integrate foreign players with local players.

The position of the team manager is crucial to raising the team’s performance and, consequently, the league’s standing in a player labor market that is liberal and marked by large migrations of international and domestic players both between clubs and between nations (Andreff, 2006). Clubs should aim to increase their playing power in the official transfer market by allowing players to move around more easily, offering greater possibilities for player development, and raising player salaries and work satisfaction (Carmichael and Thomas, 1993; Meletakos et al., 2016).

The manager of the team must weigh the club’s objectives, the league’s operating regulations, the surrounding environment, and unquestionably the preferences of the fans when making decisions because it is well known that an excessive amount of imbalance in sporting events deters fans (Kesenne, 2006) and negatively impacts the team’s present and future success. Sport migration is another result of professional and amateur sports becoming more international (Maguire and Falcous, 2010; Meletakos et al., 2016; Do Nascimento Junior et al., 2019).

It is important to critically examine the study’s conclusion, which states that adding foreign athletes to the Palestinian basketball team increases competition and boosts the team’s performance. This has a significant positive effect on the team’s resolve and morale to improve performance and make the national team. Unsupervised international player recruiting, however, may cause issues for local players, such as shorter participation times and perhaps worse player development.

On the flip side, adding international players, especially gifted ones might help the team’s native players get better. Making the best choice is what the team management and club owners need to do. The opinions of the fans, who may also be unclear in this situation, must be considered while recruiting international players. Supporters prefer to identify more with the native players and those who have been with the team for a long time, but they also think it desirable to have well-known, talented international players on their squad. When adding foreign players and considering their influence on the club, the team management needs to handle the cultural and educational difficulties that come up.

The results of this study indicate that in order to recruit new, highly skilled foreign players, the coaches of the Palestinian national volleyball team should effectively negotiate with local stadium management units. They should also use cooperative methods like naming or adopting the stadium, increasing the number of training sessions held in the home stadium, and implementing actual meaning. The team’s overall performance should be enhanced by using the nearby stadium.

The finding of this study influences both the league standings and the team’s performance. The team manager has the skills to make the best judgments by being aware of the intricate details stated above. The implications of the results include pointing out the importance of national leagues that focus on the participation of foreign players in the Palestinian National Basketball League and acknowledging the need for further studies in this area, which is relatively under-researched.

The new tool can be utilized by sports team coaches and managers to assess and improve team cohesion, especially when integrating foreign players. As highlighted by Schinke et al. (2021), well-designed tools contribute significantly to improving coaching practices and team management.

### Limitations and future research directions

Our research has some limitations. The small sample size of participants in the study is one of its limitations, which might have affected how statistically representative the findings were. To provide even more clarity, qualitative information from a few of the interviews was used. The second limitation includes the non-experimental study design and the absence of a probability sample of national team members, which restricts the applicability of the findings to other Middle Eastern countries.

From the perspective of local players, the study sought to determine how international players’ presence in the Palestinian Basketball League affected team cohesion. It is necessary to do more study that takes into account the qualitative traits of international competitors. A player’s existence and, more crucially, whether or not he is a member of his national team and has won trophies while playing for it, are examples of one of numerous traits. Whether or whether he plays for elite teams domestically or overseas, and whether or not these teams have produced championships, is another factor to examine. Another crucial criterion is whether or whether the athlete has experience playing in reputable high-level basketball leagues like the German Handball League or the NBA.

Examining the competitive balance of lower-ranked leagues within a nation may be a future study focus. These leagues often make up the pool of players that major league teams choose from when making their player selections. Thus, we may inquire about the impact of lower-tier leagues on international players’ involvement in the Palestinian Basketball League on both the competitive balance in higher-tier leagues overseas and the cohesiveness and performance of these players. Open up avenues for studies on the impact of foreign players on other dimensions and sports.

Finally, one significant performance-related element that might be investigated is player age. It is common for “star players” to enter inferior levels when they are getting close to the conclusion of their playing careers. These qualitative traits can serve as the foundation for a multifactor model that includes all of the aforementioned elements.

## Data Availability

The original contributions presented in the study are included in the article/supplementary material, further inquiries can be directed to the corresponding author.
